# Scientific Items

**Published:** 1885-06-20

**Authors:** 


### SCIENTIFIC ITEMS.

**About Snakes.**—Mr. Rheem, who has charge of the reptile specimens in the Smithsonian Institute, contradicts much of the popular belief as to snakes. Some of the most dreaded have no existence. The hoop-snake, which takes the end of its tail in its mouth, and rolls over and over like a hoop, killing everything it touches, with its venom ; and the blow-snake, the breath of which is deadly,—are fictions. As serpents move about, they are constantly feeling ahead with the tongue ; and the forward thrust and peculiar forked appearance of the organ has given rise to the false idea that with it the stinging is done. It is generally thought that there are a great number of these poisonous snakes. In North America there are but three species—the rattlesnake, the copperhead or moccasin, and the coral. There are about thirty varieties of these species, altogether. The copperhead is probably the most dangerous ; as it is vicious, and never gives warning of any kind before striking. The rattlesnake, though more poisonous than eihter of the others, will rattle at the approach of anything, and try to get away unless brought to bay. The coral is much smaller, and a native of the Southern States. The bite is not necessarily fatal if the proper remedies are used in time ; as, on account of its size, the quantity of poison is small. When a reptile strikes, he throws his whole body forward, and the fangs penetrate the object against which they come. He does not jump,—the hinder part of the body remains in position ; and none of our snakes are in the habit of reaching more than half their length.—*Pop. S. News.*

**Mercury Volatile.**—It may not be generally known, that although mercury boils at 359°, yet, like water, it is slightly volatile at all temperatures. This is very prettily shown by writing, with a quill pen on paper, with a weak solution of chloride of platinum. The writing or drawing will be nearly or quite invisible until it is held over a dish containing a little mercury, when the mercurial vapor, uniting with the platinum, will cause the image to appear in dark tints. This experiment shows very clearly the formation of mercurial vapors at ordinary temperatures, and suggests the precaution of keeping vessels containing that metal tightly closed, as instances have been known where delicate persons have been injuriously affected from this cause.—*Pop. S. News.*

**All Flame** is due to burning gas ; and, when a candle or piece of paper is burned, it is really gas that is burning, just the same as if it was issuing from a gas burner. The only difference is, that in one case the gas is distilled from coal at a distant point, and in the other it is distilled on the spot from the burning wax or paper. —*Pop. S. News.*

**Doctors Disagree.**—According to the New York *Evening Post*, of July 30, M. Pasteur entirely dissents from Dr. Koch's startling advice to discontinue the practice of watering the streets during the prevalence of the cholera epidemic. He admits that the dust may be full of microbes at such a time, and that water is their native element. But, argues M. Pasteur, if they are so dry as to have lost their vitality, water will not bring them to life again. If, on the other hand, they possess sufficient humidity to be susceptible of resuscitation and doing mischief, the water process, if the supply of the fluid is adequate, will have the effect of sweeping them into the limbo of the sewers, where they will be powerless for further evil. If they are left in the streets to be blown about by every wind, they are likely to be blown in contact with the mucous membrane of passing pedestrians as with anything else, and that offers an ideal field for the recovery and development of those of them whose vitality is not absolutely extinct. M. Pasteur's reasoning has been accepted by the French faculty.

**Case of Caudal Development in a Human Being.**—Dr. Lissner, in the last number of *Virchow's Archieves*, describes a case of complete development of a tail in the human being. The Dr. delivered a multipara of a female child, which exhibited a perfect tail. Both parents were of normal frame and their other children were not deformed. It is stated that the father was given to liquor, but it does not appear that this indulgence might have effected the reversion in type suggested by the tail. The tail was an undoubted continuation of the spine; through the more delicate integument on the anal side, several bones like the digital phalanges could be felt. A cyst was attached which was somewhat hairy; this was punctured, letting escape a serous liquid. The parents would not allow an amputation. The child is now over thirteen years old. It was lately brought before the doctor for re-examination. The child by law should attend the public school, but had obstinately refused; among other reasons because, as she declared, the sitting position caused pain. The tail at present measures in length 12.5 cm., and 23 cm. in circumference. Hard, iregular bodies, bones probably, can be felt through the skin.—*St. Louis Cour. Medicine.*

**The halo,** or "rainbow," seen round the moon, is due to the refraction of the light through minute drops of water or ice-crystals in the upper air. It indicates that there is an excess of aqueous vapor in the air, and consequently is generally seen just before a storm.—*Pop. S. News.*

**The humming** sound heard in the vicinity of telegraph wires is not due to the passage of the eclctric current, but to the vibration of the wire by the wind. The poles and wires form, in fact, a great Æolian harp.—*Pop. S. News.*

**Platinum** is not so costly a metal as gold, which is the most valuable of all, except, of course, those rare metals like erbium, lanthanum, etc., which are only chemical curiosities, and have no fixed commercial value.—*Pop. S. News.*

